# Redox-Related Genetic and Biological Ageing Signals in Rapid Pain Progression of Knee Osteoarthritis: A Hypothesis-Generating Analysis in the Osteoarthritis Initiative

**DOI:** 10.3390/antiox15020266

**Published:** 2026-02-21

**Authors:** Francisco J. Blanco, Natividad Oreiro, Jorge Vázquez-García, Antonio Morano-Torres, Sara Relaño, Laura Martínez-Sotodosos, Diana M. Noriega-Cobo, Fátima Otero-Fariña, Alejandro Mosquera, Jose L. Fernández, Ignacio Rego-Pérez

**Affiliations:** 1 Grupo de Investigación en Reumatología (GIR), Instituto de Investigación Biomédica de A Coruña (INIBIC), Complexo Hospitalario Universitario de A Coruña (CHUAC), SERGAS, Universidade da Coruña (UDC), C/As Xubias de Arriba 84, 15006 A Coruña, Spain; 2 Grupo de Investigación en Reumatología y Salud (GIR-S), Centro de Investigación de Ciencias Avanzadas (CICA), Departamento de Fisioterapia, Medicina y Ciencias Biomédicas, Facultad de Fisioterapia, Campus de Oza, Universidade da Coruña (UDC), 15008 A Coruña, Spain; 3 Laboratorio de Genética Molecular y Radiobiología, Centro Oncológico de Galicia, C/Doctor Camilo Veiras, 1, 15009 A Coruña, Spain; 4 Unidad de Genética, Instituto de Investigación Biomédica de A Coruña (INIBIC), Complexo Hospitalario Universitario de A Coruña (CHUAC), SERGAS, Universidade da Coruña (UDC), As Xubias, 15006 A Coruña, Spain

**Keywords:** knee osteoarthritis, rapid pain progression, oxidative stress, redox imbalance, mitochondrial DNA haplogroups, leukocyte telomere length, genome-wide association study, pain trajectories

## Abstract

Rapid pain progression in knee osteoarthritis (OA) is heterogeneous and may reflect redox-related mechanisms. We performed an exploratory analysis in Osteoarthritis Initiative (OAI) participants, combining nuclear genome-wide association, mitochondrial DNA (mtDNA) haplogroups, and leukocyte telomere length. Rapid pain progression was defined using the rescaled Western Ontario and McMaster Universities Osteoarthritis Index (WOMAC) for pain (0–100) within 24-month windows. An additive genome-wide association study (GWAS) in 2946 participants tested 7,762,204 imputed variants, adjusting for age, sex, body mass index (BMI) and three principal components. Haplogroups were analysed in 3357 participants, and telomere length (telomere-to-single-copy gene, T/S, ratio) was analysed in 301 participants. No variant reached genome-wide significance (*p* < 5 × 10^−8^), but six loci were suggestive (*p* < 5 × 10^−6^), with minimal inflation (λ = 0.995). mtDNA haplogroup H was nominally associated with rapid pain progression (odds ratio, OR = 1.179, *p* = 0.023). Rapid pain progressors had shorter baseline telomeres (0.825 ± 0.268 vs. 0.985 ± 0.375; *p* < 0.001), and telomere length was inversely associated with progression (OR per 1-unit T/S = 0.260, *p* = 0.007). These preliminary, hypothesis-generating findings are compatible with a redox-related interpretation of rapid pain progression and require external validation in independent cohorts, while providing candidates for future mechanistic studies.

## 1. Introduction

Osteoarthritis (OA) is a chronic and heterogeneous disease and one of the leading causes of pain and disability worldwide. In 2020, an estimated 595 million people were affected (≈7.6% of the global population), representing a marked increase since 1990 [1]. In Spain, the EPISER2016 study estimated a high prevalence of symptomatic OA, including knee OA [2]. With population ageing and the persistence of modifiable risk factors, OA prevalence is projected to increase substantially over coming decades, with major health–economic consequences due to clinical care, pharmacological treatments, and joint replacement surgery [1,3].

Although structural changes are central to OA pathophysiology, pain is the symptom that most strongly drives disability, impaired quality of life, and health-care use. The course of pain in OA is heterogeneous; while some patients maintain stable trajectories or even improve, others experience rapid and sustained progression, suggesting the existence of distinct clinical subphenotypes or patterns [4,5,6,7]. Recent work has standardised operational definitions of rapid progression using 24-month windows, improving comparability across cohorts and supporting the development of predictive models [8,9].

Genetic studies focusing on pain rather than structural damage in knee OA remain limited, but they already suggest that pain has a partly distinct genetic architecture [10,11]. Candidate-gene associations have implicated variants in *TRPV1*, *PCSK6* and *COMT* in painful versus painless knee OA, supporting nociceptive and monoaminergic contributions that are not fully explained by the radiographic severity [12,13,14]. This pain-structure discordance has also been observed in the Spanish PROCOAC cohort, including patients with KL ≤ 2 who still reported significant pain [15]. More recently, variation in *TACR1* has been associated with knee OA pain, and the SNP rs16868943 at *COL11A2* has been linked to experimental pain sensitivity: a proxy for altered pain sensitisation [16,17]. As *COL11A2* encodes a cartilage extracellular matrix collagen, this association is more likely to be indirect than a direct neuronal sensitisation mechanism. In addition, functional variation in *SCN9A* provides a clear proof of concept that germline changes in nociceptive ion channels can shape pain susceptibility [18,19]. At the polygenic level, a GWAS of knee pain in UK Biobank identified loci near *GDF5* and *COL27A1*, indicating that common variants can influence both joint damage and the propensity to report pain [20].

Mitochondrial biology offers a plausible mechanistic bridge between joint pathology and pain amplification. Mitochondria regulate cellular bioenergetics and are major sources of reactive oxygen species (ROS), which act as redox signalling mediators in inflammation and stress responses. Mitochondrial DNA (mtDNA) haplogroups have emerged as modifiers of OA phenotypes; several cohort studies and meta-analyses have shown that specific mtDNA haplogroups influence the prevalence, incidence and progression of OA, including data from the Osteoarthritis Initiative (OAI) and other populations [21,22,23,24]. These effects are consistent with experimental evidence showing that mitochondrial dysfunction, mtDNA damage and oxidative stress contribute to chondrocyte apoptosis, matrix degradation and joint inflammation [25,26,27]. Importantly, mitochondrial energetics and redox imbalance can also modulate neuroimmune pathways and nociceptor sensitisation, raising the possibility that mitochondrial DNA (mtDNA) background influences pain trajectories in knee OA [28,29].

Markers of biological ageing provide an additional layer of vulnerability that is relevant to redox biology. Leukocyte telomere length reflects cumulative exposure to oxidative stress and systemic low-grade inflammation, processes implicated in OA and in the peripheral/central sensitisation of pain pathways [30,31,32].

Combining inherited genetic variation with ageing-related biomarkers may help define a biologically meaningful subphenotype of rapid pain progression that is plausibly linked to redox imbalance and oxidative stress: processes that can modulate both joint tissue homeostasis and nociceptor sensitisation. To address this hypothesis, we performed a hypothesis-generating exploratory analysis in knee OA participants from the OAI, combining three complementary layers of information: (i) an exploratory single-cohort GWAS to screen for nuclear variants associated with rapid pain progression, (ii) an association analysis of mitochondrial DNA haplogroups as proxies of mitochondrial bioenergetics and redox balance, and (iii) in a smaller subsample, an evaluation of leukocyte telomere length as a marker of biological ageing and the cumulative oxidative/inflammatory burden. Together, these analyses were designed to identify candidate genetic and ageing-related signals that are compatible with a redox–related contribution to rapid pain progression, providing a framework for future replication and mechanistic studies.

## 2. Materials and Methods

### 2.1. Cohort Description

For this study, we used data from the OAI, a prospective multicentre cohort designed to investigate the onset and progression of knee OA, with publicly accessible longitudinal clinical data, imaging and biospecimens (https://nda.nih.gov/oai, accessed on 25 January 2026) [33]. Participants were followed annually for up to ten years, using standardised protocols to quantify changes in knee health over time, capturing the onset and progression of symptoms and functional limitations, the development and worsening of structural abnormalities, and longitudinal variation in imaging and biomarker-based indicators of knee OA, with ancillary assessments in other joints when available.

For the descriptive characterisation of the study cohort, we included the key clinical variables available in the OAI dataset. Specifically, sex, age and body mass index (BMI) were recorded at baseline for OAI participants.

All participating clinical centres complied with the Declaration of Helsinki and obtained local ethics approval. Patient consent was waived for this study because it exclusively involved the secondary use of de-identified data and coded DNA biospecimens obtained from the OAI, with no direct contact with participants. In the parent OAI study, written informed consent for participation and for the collection, storage, and future research use of participants’ data and biospecimens was obtained by the OAI under ethics approvals at the participating sites. The authors received only de-identified data and coded biospecimens through the OAI data/biospecimen access procedures and therefore did not have access to individual signed informed consent forms. No identifiable participant information is included in this manuscript, and consent for publication was not required.

This study received the favourable opinion of the ethics committee of XUNTA de Galicia (registration number 2024/074) on 19 February 2024.

### 2.2. Rapid Pain Progression Criteria

We operationalised rapid pain progression using scaled WOMAC pain scores (0–100) and standardised 24-month windows, following previously published approaches [8,9]. Participants were classified as rapid pain progressors if, in any valid 24-month window, they met at least one of the following criteria:

- Increase of at least 10 points over 24 months and substantial pain at the end of the window (WOMAC pain ≥ 40);
- Increase of at least 20 points over 24 months with end pain ≥ 35;
- Sustained substantial pain (WOMAC pain ≥ 40 at both the beginning and end of the 24-month window).

A 24-month window was considered valid if WOMAC pain scores were available at all annual visits within the window. Participants were classified as non-rapid pain progressors only if they had at least one valid observation period of 48 months or longer in which they did not meet rapid pain progression criteria in any embedded 24-month interval. This conservative control definition was used to reduce the misclassification of participants with intermittent rapid worsening or missing follow-up assessments.

### 2.3. Genetic Analyses

Genetic information for this study was obtained from two different sources: nuclear genome-wide data and mitochondrial data.

#### 2.3.1. Nuclear Genome-Wide Data

We used nuclear genome-wide genotype data generated as part of the Genetic Components of Knee Osteoarthritis (GeCKO) study [34] in OAI participants. These data had been previously deposited in dbGaP (accession phs000955.v1.p1) and were used to extract single nucleotide polymorphisms (SNPs) across the genome for association analyses.

Genotyping was performed in 4219 OAI participants, using the Illumina HumanOmni2.5 BeadChip (Illumina, San Diego, CA, USA). Quality control (QC) was conducted with PLINK 2.0 [35] and excluded single nucleotide polymorphisms (SNPs) with a low call rate, duplicates, monomorphic variants, a minor allele frequency (MAF) < 0.05 or significant deviation from the Hardy–Weinberg equilibrium (*p* < 1 × 10^−6^). At the sample level, individuals with >3% missing genotypes were removed; at the variant level, SNPs with >2% missingness were filtered out. We further excluded duplicate samples, heterozygosity outliers (>±4 SD from the mean) and ancestry outliers (>±8 SD from the European cluster in the 1000 genomes reference principal component analysis (PCA) space). Post-QC variant call format (VCF) files were imputed on the Michigan Imputation Server (Minimac4) (Ann Arbor, MI, USA), retaining variants with imputation quality (Rsq) > 0.8, yielding approximately 8 million SNPs.

#### 2.3.2. Mitochondrial Data

Mitochondrial genetic variation was captured through European mitochondrial DNA (mtDNA) haplogroups, which had been previously assigned by our group to OAI participants following a single base extension (SBE) assay-based protocol described elsewhere [21].

### 2.4. Telomere Length Assay

A subset of (n = 301) OAI participants was used to examine the influence of the baseline telomere length on the risk of rapid pain progression. The telomere length of peripheral blood leukocytes (PBLs) from this subset of patients had been previously characterised [31] using a validated quantitative PCR-based assay, as described elsewhere [36]. Briefly, this method quantifies the average ratio of the telomere repeat copy number to a single-copy reference gene (36B4) in each sample (T/S ratio) and provides an indirect estimate of the mean telomere length.

### 2.5. Statistical Analyses

Data were analysed using IBM SPSS Statistics, version 29 (IBM Corp., Armonk, NY, USA) and R software, version 4.4.1 (R Foundation for Statistical Computing, Vienna, Austria).

The genome-wide association study (GWAS) was performed using PLINK 2.0, for the post-imputation processing, and rvtests [37], for the association analyses under an additive genetic model, adjusting for age, sex, body mass index (BMI) and the first three principal components of ancestry derived from PCA on a pruned set of hard-called SNPs using stricter missingness and relatedness thresholds. The final dataset comprised 2946 QC-passed OAI participants of European ancestry and 7,762,204 SNPs, with rapid pain progression (yes/no) as the outcome. Genome-wide significance was defined as *p* < 5 × 10^−8^. To summarise the results and prioritise independent association signals, we applied linkage disequilibrium (LD)-based clumping in PLINK 2.0 (r^2^ > 0.2 within the study sample), retaining the lead SNP with the lowest *p*-value in each linkage disequilibrium cluster. Lead variants with *p* < 5 × 10^−6^ were considered suggestive and were reported as hypothesis-generating candidates. Exploratory regulatory annotation of the lead variants was performed using FORGGE 2.0 (Roadmap 15-state chromatin models and H3 marks) and RegulomeDB v2.2.

Haplogroup and allele frequencies between rapid pain progressors and non-rapid pain progressors were compared using chi-square tests from contingency tables. Odds ratios (ORs) and 95% confidence intervals (CIs) were calculated to assess the odds of carrying each allele in rapid pain progressors compared with non-progressors. For the haplogroup analysis, we followed a previously described approach, comparing each haplogroup with all remaining haplogroups pooled into a single reference category. In addition, a regression model adjusting for the confounder variables of sex, age and BMI was constructed to assess the independent associations between mtDNA haplogroups and rapid pain progression.

PBL telomere length (T/S ratio) was first compared between rapid and non-rapid pain progressors, using the non-parametric Mann–Whitney U test. We then performed a logistic regression, adjusting for sex, age and BMI, to evaluate PBL telomere length as a potential risk factor associated with rapid pain progression.

Forest plots were generated to summarise effect sizes (OR) and 95% CIs across the three genetic layers: lead nuclear GWAS loci, mtDNA haplogroups and leukocyte telomere length.

### 2.6. Declaration of Generative AI and AI-Assisted Technologies in the Writing Process

During the preparation of this work the author(s) used ChatGPT 5.2 in order to adjust the different R scripts. After using this tool, the author(s) reviewed and edited the content as needed and take(s) full responsibility for the content of the publication.

## 3. Results

### 3.1. Study Population

The complete dataset included a total of 3395 OAI participants of European ancestry, of which 1906 (56.1%) were women and 1489 (43.9%) were men. Overall, 1260 (37.1%) participants met the criteria for rapid pain progression and 2135 (62.9%) were classified as non-rapid pain progressors. A descriptive analysis of this population showed that rapid pain progressors were more likely to be female (*p* < 0.001) and to have a higher BMI (*p* < 0.001) than non-rapid pain progressors (Table 1).

### 3.2. Nuclear Genome-Wide Analysis

**Figure 1:**
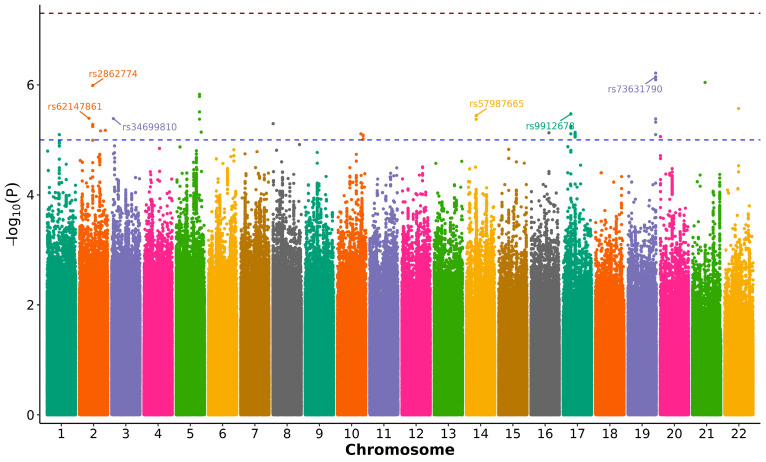
Manhattan plot of the genome-wide association study (GWAS) for rapid pain progression in knee osteoarthritis. Association results for 7,762,204 imputed SNPs tested in OAI participants of European ancestry under an additive genetic model, adjusting for age, sex, BMI and the first three ancestry principal components. Each point represents an SNP (y-axis: −log_10_(*p*)) plotted by chromosomal position (x-axis; alternating colours by chromosome). The red dashed line indicates the conventional genome-wide significance threshold (*p* = 5 × 10^−8^), and the blue dashed line indicates the suggestive threshold (*p* = 5 × 10^−6^). No variant reached genome-wide significance; six loci exceeded the suggestive threshold and were carried forward as hypothesis-generating candidates (Table 2).

We conducted a GWAS to identify nuclear genetic variants associated with rapid pain progression. As described in Section 2, the final GWAS dataset included 2946 QC-passed OAI participants of European ancestry and 7,762,204 imputed SNPs, with rapid pain progression (yes/no) as the outcome. Overall, the GWAS showed no evidence of substantial test statistic inflation (genomic inflation factor λ = 0.995), and the quantile–quantile (QQ) plot suggested adequate control of population stratification and relatedness (Supplementary Figure S1). PCA plots and PC-phenotype association results are provided in Supplementary Figure S2 and Supplementary Table S1, respectively. The Manhattan plot is shown in Figure 1.

**Figure 2:**
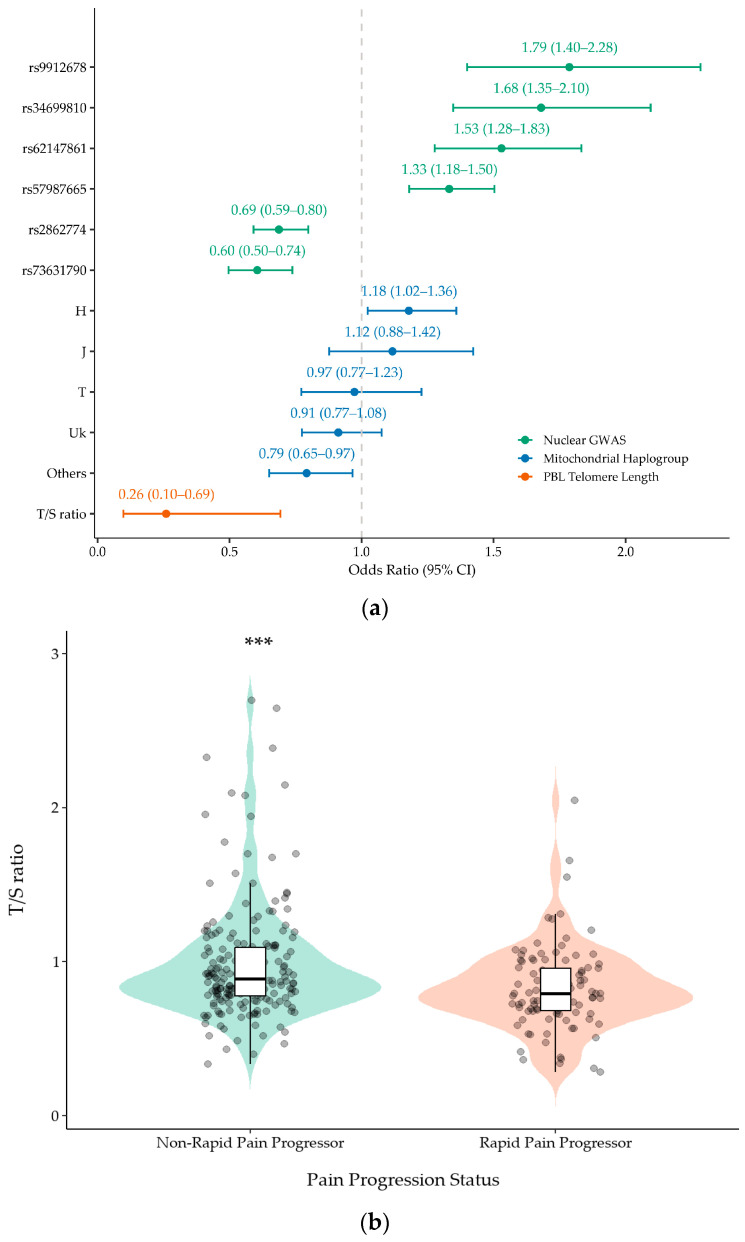
Effect sizes across genetic layers and distribution of leukocyte telomere length. (**a**) Forest plot showing odds ratios (ORs) and 95% confidence intervals for the six lead suggestive nuclear GWAS variants (*p* < 5 × 10^−6^), mitochondrial haplogroups, and peripheral blood leukocyte (PBL) telomere length (T/S ratio) in relation to rapid pain progression. Points indicate effect estimates and horizontal bars indicate 95% CIs; the vertical dashed line marks the null (OR = 1). (**b**) Violin/box plot of baseline leukocyte telomere length (T/S ratio) by pain progression status. Violin widths represent the distribution density; boxes show the median and interquartile range (IQR), with whiskers extending to 1.5 × IQR; points represent individual participants. *** indicates *p* < 0.001 for the between-group comparison.

No SNP reached the conventional threshold for genome-wide significance (*p* < 5 × 10^−8^). However, we identified six independent loci that met the predefined suggestive significance threshold (*p* < 5 × 10^−6^) and were therefore considered candidate signals for further evaluation (Table 2). The strongest association signal was observed for rs73631790 on chromosome 19q13.43 (*p* = 7.195 × 10^−7^), located in the proximal 5′ flanking region of *DUXA*, a double homeobox transcription factor gene. Another prominent signal, rs2862774 on chromosome 2q14.1 (*p* = 1.03 × 10^−6^), maps intronically within *IL36B*, which encodes the interleukin-36β cytokine. Among the remaining loci, rs9912678 on 17p11.2 (*p* = 3.37 × 10^−6^) lies in the upstream flanking region of *RAI1*; rs57987665 on 14q21.3 (*p* = 3.62 × 10^−6^) falls in an intergenic region, with the closest annotated transcript being the long non-coding RNA LOC105378178; rs34699810 on 3p26.1 (*p* = 4.11 × 10^−6^) is intergenic and nearest to the antisense lncRNA LMCD1-AS1; and rs62147861 on chromosome 2 (*p* = 4.06 × 10^−6^) is located in proximity to the uncharacterised non-coding RNA LOC124907827. The effect sizes for these variants were modest to moderate (Table 2) (Figure 2a), as expected for complex traits. In addition, the exploratory in silico regulatory enrichment/annotation of these lead loci is summarised in Supplementary Table S2.

The exploratory functional annotation of these six lead variants using GTEx Portal is summarised in Supplementary Table S3. Briefly, rs9912678 shows a cis-eQTL signal for *ATPAF2* (skeletal muscle) and an sQTL for *RAI* (cultured fibroblasts); rs57987665 shows an sQTL for *KLDC1* (oesophagus mucosa); and rs2862774 shows cis-eQTL signals for *IL36B/IL1A* (skin, spleen) and sQTL signals for *IL37/PSD4* (skin, pancreas).

Given the limited sample size and the absence of genome-wide significance findings, these association signals should be interpreted as exploratory and hypothesis-generating. Replication in independent cohorts and functional follow-up will be required to confirm their role in rapid pain progression.

### 3.3. Mitochondrial DNA Analysis

Haplogroup data were available for 3357 OAI participants, of which 1239 (36.91%) were rapid pain progressors and 2118 (63.09%) were non-rapid pain progressors. The frequency distribution of major European mtDNA haplogroups ranged from 9% for haplogroup J to 40.9% for the most common haplogroup H (Table 3). In unadjusted analyses, only mtDNA haplogroup H showed a nominally significant over-representation in the rapid pain progressors group (43.4%) compared with non-rapid progressors (39.4%) (OR = 1.179; 95%CI = 1.023–1.359; *p* = 0.023); however, this association did not remain significant after Bonferroni correction (q-value = 0.115). In addition, the pooled “other” haplogroup category (individual haplogroup frequency <5%) was nominally associated with a lower risk of rapid pain progression (OR = 0.792; 95%CI = 0.650–0.966; *p* = 0.021); however, given its heterogeneous composition, this finding was considered exploratory and was not interpreted further. No other haplogroup showed a statistically significant difference in frequency (Table 3) (Figure 2a).

In multivariable logistic regression, carriers of haplogroup H remained more likely to be rapid pain progressors (OR = 1.172; 95%CI = 1.012–1.357; *p* = 0.034), and rapid pain progression was also independently associated with the female sex (OR = 1.494; 95%CI = 1.289–1.732; *p* < 0.001), age (OR = 1.009; 95%CI = 1.001–1.017; *p* = 0.031) and higher BMI (OR = 1.113; 95%CI = 1.095–1.131; *p* < 0.001) (Table 4).

### 3.4. Telomere Length Analysis

In the subset of 301 OAI participants with available PBL telomere measurements, 103 (34.22%) were classified as rapid pain progressors and 198 (65.78%) as non-rapid pain progressors. The baseline PBL telomere length (T/S ratio) was 0.825 ± 0.268 in rapid pain progressors and 0.985 ± 0.375 in non-rapid pain progressors (*p* < 0.001, Mann–Whitney U test) (Figure 2b). In multivariable logistic regression adjusting for sex, age and BMI, the PBL telomere length was inversely associated with rapid pain progression (OR per 1-unit increase in T/S = 0.260; 95%CI = 0.098–0.693; *p* = 0.007) (Table 5) (Figure 2a).

## 4. Discussion

This hypothesis-generating study aimed to investigate redox-linked genetic and biological ageing determinants of rapid pain progression in knee OA, using participants from the OAI. Using a standardised 24-month window definition, we combined a discovery GWAS with mtDNA haplogroup profiling, and additionally evaluated the baseline PBL telomere length in a subset of participants. As an exploratory GWAS without external replication or meta-analysis, these findings should be interpreted cautiously: no variants reached the conventional genome-wide significance, but six suggestive loci were identified as hypothesis-generating candidates. In parallel, mtDNA haplogroup H was nominally over-represented among rapid pain progressors, and a shorter baseline telomere length was associated with increased odds of rapid pain progression after adjustment for sex, age and BMI.

To our knowledge, none of the six lead SNPs identified in this study have been previously reported as being associated with OA or pain phenotypes, nor do they overlap with the established set of genome-wide significant loci for structural OA described in recent large-scale meta-analyses [11,38,39,40]. Notably, our GWAS focused on a strictly defined longitudinal phenotype of rapid pain progression, whereas most prior OA pain genetics have examined cross-sectional symptomatic status or broader pain traits; together with limited power in a single-cohort design, this may partly explain the lack of overlap with previously reported loci.

To provide exploratory functional context for the lead loci, we queried the GTEx Portal for cis-eQTL and sQTL signals. Because GTEx does not include articular cartilage or synovium, these annotations are presented as supportive context in non-joint tissues and do not establish mechanisms in OA-relevant joint tissues. As additional regulatory context, we also performed exploratory in silico enrichment/annotation, which suggested that several lead loci map to active regulatory features in bone- and immune-related cellular contexts, with rs9912678 and rs2862774 prioritised as the strongest regulatory candidates. However, these annotations are based on public resources and remain supportive and hypothesis-generating, rather than demonstrating tissue-specific mechanisms in OA-relevant joint tissues. At the gene level, *IL36B* encodes interleukin-36β, a member of the IL-1 cytokine family that stimulates the production of *IL-6*, *IL-8* and matrix metalloproteinases by synovial fibroblasts and articular chondrocytes, and *IL-36* family cytokines are increasingly recognised as mediators of joint inflammation [41,42,43]. Moreover, spinal *IL-36γ*/*IL-36R* signalling has been shown to participate in the maintenance of chronic inflammatory pain in preclinical models, supporting a plausible link between this locus and pathways of inflammatory nociception [43,44]. Notably, *IL-36* signalling has also been shown to modulate glutathione homeostasis and reactive oxygen species (ROS) resolution in experimental systems, suggesting a potential mechanistic bridge between inflammatory activation and redox imbalance [45]. However, in the absence of locus-specific functional evidence, this remains speculative.

*RAI1*, the nearest gene to the 17p11.2 signal, is the dosage-sensitive transcription factor responsible for Smith–Magenis syndrome, a neurodevelopmental disorder characterised by altered temperature and pain sensitivity, and the *RAI1* dosage modulates pain sensitivity in mouse models [46,47]; in addition, *RAI1* has recently been prioritised as a putative effector gene at an all-OA locus in integrative analyses that combine OA GWAS with osteoclast regulatory data [48]. Beyond nociception-related phenotypes, patient-derived and cellular models of *RAI1* haploinsufficiency have reported both mitochondrial and autophagy alterations accompanied by increased oxidative stress/ROS signatures, which is consistent with, but does not establish, a redox-related hypothesis for this locus [49]. In GTEx, rs9912678 shows cis-eQTL and sQTL signals in non-joint tissues.

By contrast, *DUXA* is a paralogue of *DUX4*, an embryonic transcription factor that is causally involved in facioscapulohumeral muscular dystrophy, and although members of the *DUX* family are important regulators of early myogenic programmes, no direct role of *DUXA* in OA or pain has yet been reported [50,51]. However, DUX4 has been tightly connected to oxidative stress biology in muscle, including evidence that oxidative stress can upregulate *DUX4* expression and that *DUX4*-related phenotypes involve ROS/DNA damage pathways that can be mitigated by antioxidant strategies, providing an indirect redox-relevant context for this gene family [52].

The remaining loci map to poorly characterised long non-coding RNAs: LMCD1-AS1, which has been implicated as an oncogenic lncRNA in osteosarcoma, cervical and thyroid cancers, and the uncharacterised ncRNAs LOC105378178 and LOC124907827, for which no specific function in musculoskeletal disease or nociceptive pathways is currently known [53].

Taken together, our findings did not identify definitive genome-wide significant variants for rapid pain progression, but they highlighted a small set of biologically plausible candidate loci, particularly at *IL36B* and *RAI1*, with potential links to inflammatory–redox mechanisms that merit further investigation. In line with the single-cohort design and the use of a suggestive significance threshold, these association signals should be regarded as exploratory and primarily hypothesis-generating, and replication in independent cohorts and functional studies will be required to confirm whether these loci contribute to the genetic architecture of rapid pain progression in knee OA.

Our finding that mtDNA haplogroup H was nominally over-represented among rapid pain progressors is consistent with a growing body of evidence indicating that mitochondrial genetic background can modulate OA phenotypes. In European-descent populations, mtDNA haplogroups have been linked to knee OA prevalence and severity, as well as to disease incidence and progression across cohorts including the OAI, supporting the concept that mtDNA lineages may influence joint vulnerability beyond traditional risk factors [21,23,54,55]. Mechanistically, mtDNA haplogroups differ in oxidative phosphorylation performance and redox balance; haplogroup H has been associated with higher oxygen consumption and oxidative damage compared with haplogroups such as J, which has been discussed as a more uncoupled/low-ROS background in OA-related contexts [22,56]. Given the established links between mitochondrial dysfunction/oxidative stress and neuroimmune signalling in pain sensitisation, mtDNA haplogroups could plausibly contribute not only to structural trajectories but also to pain trajectories in knee OA [28,57]. Nevertheless, because the observed association is nominal and the “Other” haplogroup category aggregates heterogeneous lineages, these results should be interpreted cautiously, and they require replication in independent datasets with comparable longitudinal pain phenotyping.

In the telomere sub-study, rapid pain progressors exhibited shorter baseline leukocyte telomere length, supporting the notion that biological ageing and cumulative systemic stress may modulate symptomatic worsening in knee OA. Prior work in the OAI has linked shorter leukocyte telomere length to radiographic knee OA and greater structural burden, and longitudinal analyses have further suggested that accelerated telomere attrition is associated with incident knee OA during follow-up [31,58]. Importantly, telomere length has also been related to the biological burden of chronic knee OA pain: individuals with higher chronic pain severity, and/or high perceived stress in the context of chronic knee OA pain, show shorter telomeres, which is consistent with an accelerated ageing signal in more severe symptomatic presentations [30,59]. Conceptually, telomere length is an integrative marker of biological ageing, influenced by oxidative and inflammatory stressors that are relevant to OA pathophysiology and pain sensitisation [32]. While our results align with this framework, they derive from a subset of patients and, therefore, warrant cautious interpretation and replication, ideally in cohorts with repeated telomere measures and harmonised longitudinal pain phenotypes.

This study has several limitations. First, the genome-wide component was conducted in a single discovery cohort without external replication or meta-analysis, and no associations reached conventional genome-wide significance; therefore, the identified loci should be regarded as exploratory and hypothesis-generating. Direct in silico replication was not feasible because comparable, harmonised longitudinal pain trajectories are not available in major public resources; thus, our top signals remain preliminary and require external validation in independent cohorts. Finally, the sample size is modest for a GWAS of this complex phenotype, and the lack of genome-wide significant findings may reflect limited power, rather than the absence of genetic effects. In addition, oxidative stress was not directly measured and no experimental epigenomic or proteomic profiling was performed; our regulatory evidence is limited to exploratory in silico annotation, so mechanistic inferences remain hypothesis-generating. Second, although we used a strict and clinically meaningful definition of rapid pain progression, misclassification remains possible, given the inherent variability of self-reported pain and the influence of time-varying factors such as analgesic use, intra-articular treatments and comorbidities. Third, the telomere analysis was restricted to a relatively small subset of participants, which limits power and may introduce selection bias, and the “other” mtDNA haplogroup category aggregates heterogeneous lineages, reducing their biological interpretability. As contingency measures, we report effect sizes with confidence intervals, emphasise internal consistency across complementary genetic layers (nuclear GWAS, mtDNA haplogroups and telomere length), and interpret all signals cautiously within an explicitly exploratory framework. Future work will prioritise replication in independent cohorts with harmonised longitudinal pain phenotyping and, where possible, repeated telomere measurements and functional follow-up to strengthen causal inference and clarify the underlying mechanisms.

## 5. Conclusions

**Figure 3:**
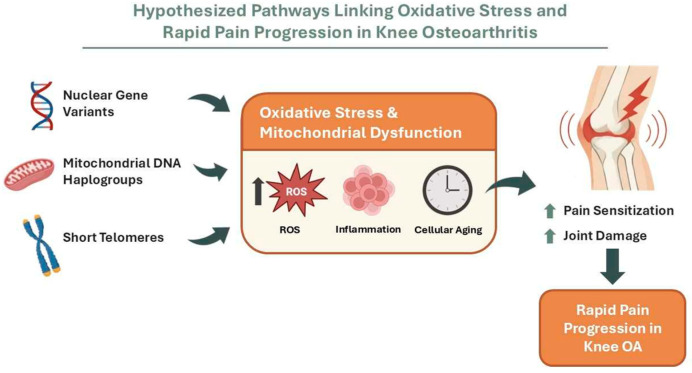
Hypothesised pathways linking oxidative stress to rapid pain progression in knee osteoarthritis in a hypothesis-generating analysis. Conceptual framework combining the three biological layers evaluated in this study—nuclear genetic variation (exploratory GWAS signals), mtDNA haplogroups, and leukocyte telomere length as a marker of biological ageing—converging on oxidative stress and mitochondrial dysfunction (increased ROS), inflammation, and cellular ageing. These processes are proposed to promote pain sensitisation and joint damage, ultimately contributing to rapid pain progression in knee OA. Image generated using artificial intelligence.

This hypothesis-generating analysis (Figure 3) suggests that rapid pain progression in knee OA may be associated with biological signals that are compatible with a redox-related hypothesis, spanning nuclear immune–neuroimmune genetic variation (including a suggestive *IL36B* signal), mitochondrial genetic background, and systemic biological ageing indexed by leukocyte telomere length. Together, these findings provide hypothesis-generating leads for replication and mechanistic studies; however, as oxidative stress was not directly measured and the genome-wide component lacked external replication, independent validation, functional follow-up and integrative multi-omics studies will be required to confirm these associations in independent cohorts and to identify actionable redox and mitochondrial targets for preventing rapid pain progression.

## Figures and Tables

**Table 1:** Descriptive analysis of the OAI participants, based on their pain progression status.

	Rapid Pain Progressors (n = 1260)	Non-Rapid Pain Progressors (n = 2135)	*p*-Value
Age at baseline (years)	61.82 ± 9.05	61.33 ± 9.32	0.116 *
Sex:			**<0.001 ^#^**
Male	494 (39.2)	995 (46.6)	
Female	766 (60.8)	1140 (53.4)	
BMI (kg/m^2^)	29.43 ± 4.90	27.25 ± 4.31	<0.001 *

Values are mean ± standard deviation or number of patients with percentage in parentheses; (*) Mann–Whitney U test; (^#^) chi-square test; BMI: body mass index; significant *p*-values are in bold.

**Table 2:** Suggestive associations with rapid pain progression in the discovery GWAS *.

rsID	Chr.	Ref.	Alt.	Nearest Gene	OR (95% CI)	*p*-Value
rs73631790	19	G	A	DUXA	0.605 (0.496–0.738)	7.195 × 10^−7^
rs9912678	17	A	T	RAI1	1.787 (1.400–2.284)	3.374 × 10^−6^
rs57987665	14	A	G	LOC105378178	1.332 (1.180–1.503)	3.618 × 10^−6^
rs34699810	3	A	G	LMCD1-AS1	1.680 (1.347–2.095)	4.107 × 10^−6^
rs2862774	2	A	C	IL36B	0.687 (0.591–0.798)	1.027 × 10^−6^
rs62147861	2	C	A	LOC124907827	1.530 (1.277–1.833)	4.059 × 10^−6^

(*) Adjusted for age, sex, body mass index and first 3 principal components; Chr.: chromosome; Ref.: reference; Alt.: alternative allele; OR: odds ratio; and CI: confidence interval.

**Table 3:** Frequencies and odds ratios (ORs) of mitochondrial DNA (mtDNA) haplogroups in rapid pain progressors.

Haplogroups	Rapid Pain Progressors	Non-Rapid Pain Progressors	Total		95% CI			
	(n = 1239)	(n = 2118)	(n = 3357)	OR	Lower CI	Upper CI	*p*-Value	q-Value ^&^
H	538 (43.4)	835 (39.4)	1373 (40.9)	1.179	1.023	1.359	**0.023 ^#^**	0.115
Uk	287 (23.2)	526 (24.8)	813 (24.2)	0.912	0.774	1.076	0.275	1.000
T	125 (10.1)	219 (10.3)	344 (10.2)	0.973	0.772	1.227	0.817	1.000
J	119 (9.6)	184 (8.7)	303 (9.0)	1.117	0.877	1.423	0.371	1.000
Others *	170 (13.7)	354 (16.7)	524 (15.6)	0.792	0.650	0.966	**0.021 ^#^**	0.105

Values are number of patients with percentage in parentheses: (*) the group “others” includes mtDNA lineages with a population frequency below 5%; (^#^) Chi-square test; statistical significance declared at *p* ≤ 0.05 (in bold); (^&^) q-values denote Bonferroni adjusted *p*-values correcting for multiple comparisons across the 5 haplogroups tested; OR: odds ratio; and CI: confidence interval.

**Table 4:** Multivariable logistic regression model to predict the influence of mtDNA haplogroup H on the risk of rapid pain progression in participants belonging to the OAI.

Variable	B	Adjusted OR	95% CI	*p*-Value
Sex (female)	0.402	1.494	1.289–1.732	**<0.001**
Age	0.009	1.009	1.001–1.017	**0.031**
BMI	0.107	1.113	1.095–1.131	**<0.001**
Haplogroup H	0.159	1.172	1.012–1.357	**0.034**

B: regression coefficient; OR: odds ratio; BMI: body mass index; OAI: Osteoarthritis Initiative; and CI: confidence interval. Statistical significance declared at *p* < 0.05 (in bold).

**Table 5:** Multivariable logistic regression model to predict the influence of PBL telomere length on the risk of rapid pain progression in a subset of 301 participants belonging to the OAI.

Variable	B	Adjusted OR	95% CI	*p*-Value
Sex (female)	0.140	1.150	0.688–1.923	0.593
Age	0.012	1.012	0.982–1.042	0.449
BMI	0.132	1.141	1.076–1.211	**<0.001**
T/S ratio *	−1.347	0.260	0.098–0.693	**0.007**

B: regression coefficient; OR: odds ratio; PBL: peripheral blood leukocyte; BMI: body mass index; OAI: Osteoarthritis Initiative; CI: confidence interval; and (*) average ratio of telomere repeat copy number to a single-copy reference gene (36B4) in each sample. Statistical significance declared at *p* < 0.05 (in bold).

## Data Availability

Publicly available datasets were analysed in this study. OAI data can be accessed via https://nda.nih.gov/oai (accessed on 25 January 2026). Dataset available on request from the authors.

## Supplementary Materials

The following supporting information can be downloaded at: https://www.mdpi.com/article/10.3390/antiox15020266/s1, Figure S1: Quantile–quantile (QQ) plot of GWAS p-values for rapid pain progression in knee osteoarthritis. Observed −log10(P) values are plotted against those expected under the null hypothesis for 7,762,204 imputed SNPs tested in OAI participants of European ancestry under an additive model adjusted for age, sex, BMI and the first three ancestry principal components. The close agreement with the null line indicates adequate control of population stratification and relatedness, with minimal test statistic inflation (genomic inflation factor λ = 0.995); deviation is confined to the extreme tail, consistent with a small number of suggestive association signals; Figure S2: Principal component analysis (PCA) of the GWAS sample. The scree plot (top left) shows the percentage of variance explained by the first 10 principal components (PCs). The remaining panels display pairwise PCA scatter plots (PC1 vs. PC2, PC3 vs. PC4, PC5 vs. PC6, PC7 vs. PC8, and PC9 vs. PC10). Each point represents one participant and is coloured by rapid pain progression status (as coded in the dataset; groups 1—non-rapid pain progressors- and 2—rapid pain progressors-). The substantial overlap between groups across PCs indicates no obvious residual population stratification; Table S1: Association analysis of 10 PCs with the rapid pain progression phenotype; Table S2a: FORGE 2.0 enrichment results using Roadmap Epigenomics 15-state chromatin models for lead GWAS variants; Table S2b: FORGE 2.0 enrichment results for histone H3 marks (Roadmap/ENCODE) for lead GWAS variants; Table S2c: RegulomeDB v2.2 functional annotation and regulatory ranking of lead GWAS variants; Table S3: GTEx functional annotation of lead GWAS variants.

## Abbreviations

The following abbreviations are used in this manuscript:

BMI	Body mass index
CI	Confidence interval
COL11A2	Collagen type XI alpha 2 chain
COL27A1	Collagen type XXVII alpha 1 chain
COMT	Catechol-O-methyltransferase
dbGaP	Database of Genotypes and Phenotypes
DNA	Deoxyribonucleic acid
DUX4	Double homeobox 4
DUXA	Double homeobox A
eQTL	Expression Quantitative Trait Locus
GDF5	Growth differentiation factor 5
GeCKO	Genetic Components of Knee Osteoarthritis
GTEx	Genotype-Tissue Expression
GWAS	Genome-wide association study
HWE	Hardy–Weinberg equilibrium
IL-1	Interleukin-1
IL-6	Interleukin-6
IL-8	Interleukin-8
IL-36	Interleukin-36
IL-36γ	Interleukin-36 gamma
IL-36R	Interleukin-36 receptor
IL36B	Interleukin 36 beta
IQR	Interquartile range
LD	Linkage disequilibrium
LMCD1-AS1	LMCD1 antisense RNA 1
lncRNA	Long non-coding RNA
LOC105378178	Uncharacterised non-coding RNA locus
LOC124907827	Uncharacterised non-coding RNA locus
MAF	Minor allele frequency
Minimac4	Minimac4 imputation software
mtDNA	Mitochondrial DNA
OA	Osteoarthritis
OAI	Osteoarthritis Initiative
OR	Odds ratio
PBL	Peripheral blood leukocytes
PCA	Principal component analysis
PC	Principal component
PCR	Polymerase chain reaction
QC	Quality control
QQ	Quantile–quantile
RAI1	Retinoic acid induced 1
ROS	Reactive oxygen species
r^2^	Squared correlation coefficient
RNA	Ribonucleic acid
Rsq	Imputation quality metric
SBE	Single base extension
SCN9A	Sodium voltage-gated channel alpha subunit 9
SD	Standard deviation
SNP	Single nucleotide polymorphism
SPSS	Statistical Package for the Social Sciences
sQTL	Splicing quantitative trait locus
TACR1	Tachykinin receptor 1
TRPV1	Transient receptor potential vanilloid 1
VCF	Variant call format
WOMAC	Western Ontario and McMaster Universities Osteoarthritis Index
