# Supplementary material for: Redox-Related Genetic and Biological Ageing Signals in Rapid Pain Progression of Knee Osteoarthritis: A Hypothesis-Generating Analysis in the Osteoarthritis Initiative
Source: Antioxidants (Basel). 2026 Feb 21;15(2):266. doi: 10.3390/antiox15020266 (PMC12938837; doi:10.3390/antiox15020266)
Supplement: Supplementary file 1 [file antioxidants-15-00266-s001.zip › Figure Caption S1,2.pdf]

Figure S1. Quantile–quantile (QQ) plot of GWAS p-values for rapid pain progression in knee osteoarthritis. Observed −log10(P) values are plotted against those expected under the null hypothesis for 7,762,204 imputed SNPs tested in OAI participants of European ancestry under an additive model adjusted for age, sex, BMI and the first three ancestry principal components. The close agreement with the null line indicates adequate control of population stratification and relatedness, with minimal test statistic inflation (genomic inflation factor λ = 0.995); deviation is confined to the extreme tail, consistent with a small number of suggestive association signals.

Figure S2: **Principal component analysis (PCA) of the GWAS sample**. The scree plot (top left) shows the percentage of variance explained by the first 10 principal components (PCs). The remaining panels display pairwise PCA scatter plots (PC1 vs PC2, PC3 vs PC4, PC5 vs PC6, PC7 vs PC8, and PC9 vs PC10). Each point represents one participant and is coloured by rapid pain progression status (as coded in the dataset; groups 1 –non-rapid pain progressors- and 2 –rapid pain progressors-). The substantial overlap between groups across PCs indicates no obvious residual population stratification.
