# Supplementary figures and images for: Redox-Related Genetic and Biological Ageing Signals in Rapid Pain Progression of Knee Osteoarthritis: A Hypothesis-Generating Analysis in the Osteoarthritis Initiative
Source: Antioxidants (Basel). 2026 Feb 21;15(2):266. doi: 10.3390/antiox15020266 (PMC12938837; doi:10.3390/antiox15020266)

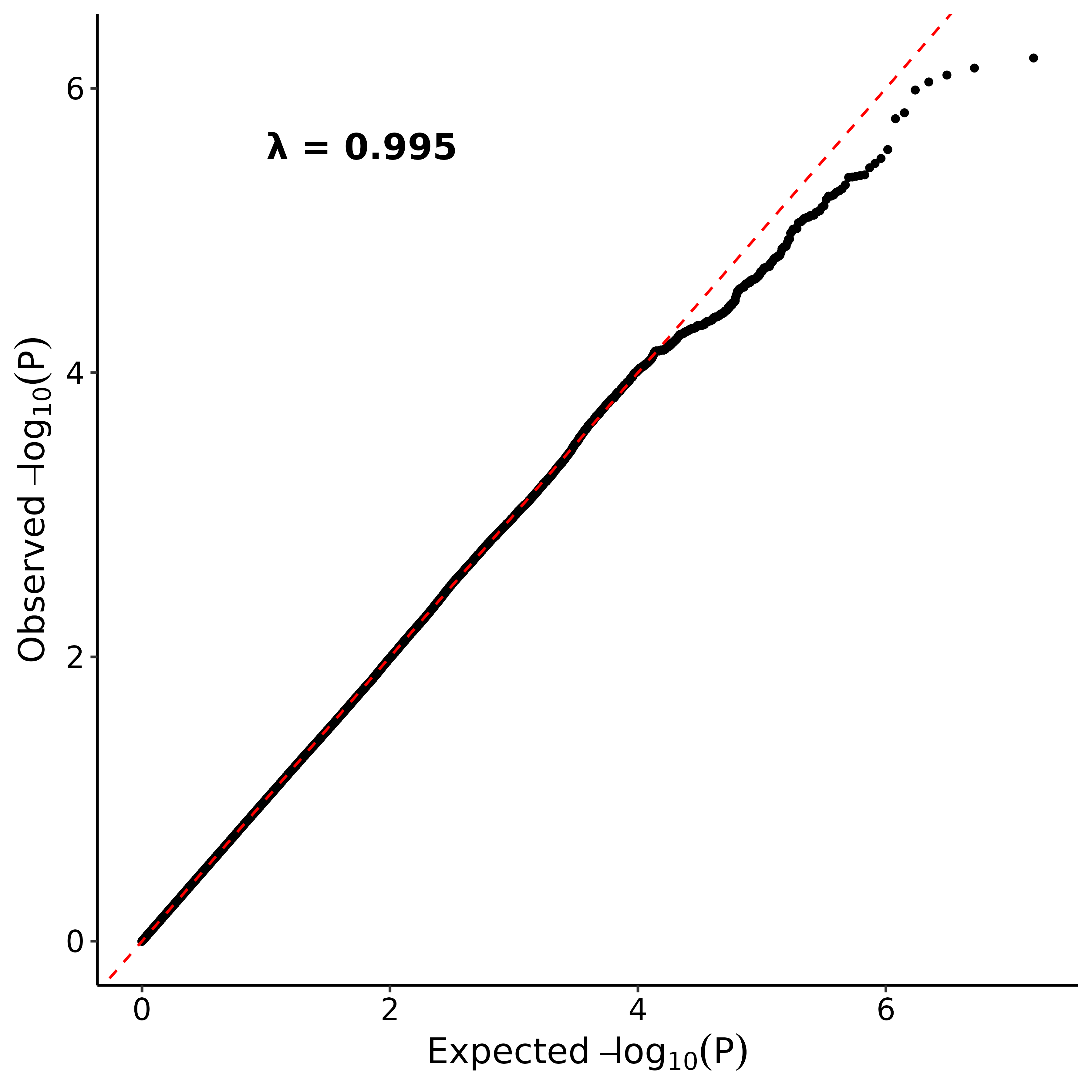

Supplement: Supplementary file 1 [file antioxidants-15-00266-s001.zip › Supplementary figure S1.tiff]

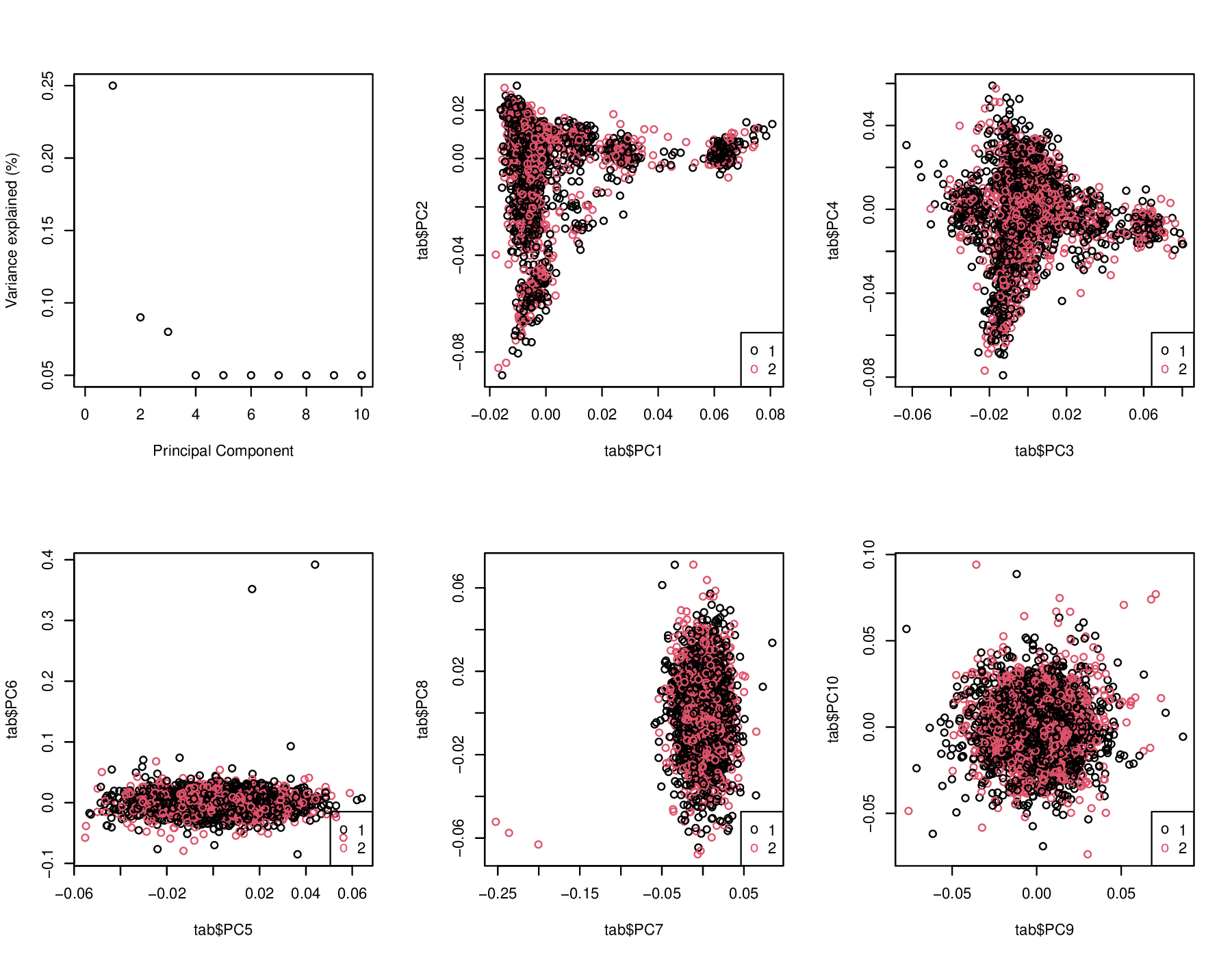

Supplement: Supplementary file 1 [file antioxidants-15-00266-s001.zip › Supplementary figure S2.tiff]
